# Renoprotective effects of crocin against colistin-induced nephrotoxicity in a rat model

**DOI:** 10.22038/IJBMS.2023.72808.15843

**Published:** 2024

**Authors:** Reza Rajabalizadeh, Mahboobeh Ghasemzadeh Rahbardar, Bibi Marjan Razavi, Hossein Hosseinzadeh

**Affiliations:** 1 Department of Pharmacodynamics and Toxicology, School of Pharmacy, Mashhad University of Medical Sciences, Mashhad, Iran; 2 Pharmaceutical Research Center, Pharmaceutical Technology Institute, Mashhad University of Medical Sciences, Mashhad, Iran; 3 Targeted Drug Delivery Research Center, Pharmaceutical Technology Institute, Mashhad University of Medical Sciences, Mashhad, Iran

**Keywords:** Anti-oxidants, Blood urea nitrogen, Creatinine, Carotenoids, Glutathione, Kidney, Malondialdehyde

## Abstract

**Objective(s):**

Colistin is used to treat multidrug-resistant gram-negative bacterial infections. It increases the membrane permeability of kidney cells, leading to kidney toxicity. Crocin, a carotenoid found in saffron, has anti-oxidant and nephroprotective properties. The present study aimed to explore the potential renoprotective effects of crocin against colistin-induced nephrotoxicity.

**Materials and Methods:**

Six groups of male Wistar rats were utilized: 1- Control (0.5 ml of normal saline, 10 days, IP); 2- Crocin (40 mg/kg, 10 days, IP); 3-Colistin (23 mg/kg, 7 days, IP); 4-6 Colistin (23 mg/kg, 7 days, IP)+ crocin (10, 20, 40 mg/kg, 10 days, IP). On day 11, rats were sacrificed and their blood and kidney samples were collected to measure creatinine, blood urea nitrogen (BUN), glutathione (GSH) levels, malondialdehyde (MDA), and histopathological alterations.

**Results:**

Colistin caused a significant increase in BUN, creatinine, and MDA, and a decrease in GSH compared to the control group. It also led to congested blood vessels, glomerular shrinkage, and medullary tubular degeneration. Co-administration of crocin with colistin resulted in a significant decrease in BUN and creatinine, increased GSH levels, and ameliorated the histopathological alterations compared to the colistin group. No significant difference was found between the control group and the crocin (40 mg/kg) group.

**Conclusion:**

It might be suggested that colistin can induce kidney damage by inducing oxidative stress. However, crocin shows protective effects against colistin-induced renal injury by acting as an anti-oxidant. Hence, crocin can be used as a supplement to reduce tissue and biochemical damage caused by colistin injection.

## Introduction

The antibacterial compound colistin, commonly known as polymyxin E, is an antibiotic and has a cationic polypeptide structure. Multidrug-resistant gram-negative bacterial infections caused by *Pseudomonas aeruginosa*, *Klebsiella pneumoniae*, and *Acinetobacter baumannii* can be effectively treated with this substance, which possesses both bactericidal and anti-endotoxic properties (1). Its mechanism of action involves the disruption of the bacterial cell membrane, which ultimately results in the death of the bacterial cells. Although it had been commercially available since 1950, it was taken off the market in the 1970s because of high rates of nephrotoxicity (2). Nephrotoxicity can occur even at therapeutic doses, and the risk of this side effect increases with higher doses and prolonged treatment (3). In the last ten years, colistin has been reintroduced as a last-resort treatment for multidrug-resistant infections, which have a notably high fatality rate in critically ill patients due to the rarity of newly discovered antibiotics (4). Colistin-induced nephrotoxicity also shows elevated blood urea nitrogen (BUN) and creatinine levels in rodents (5). One of thee main mechanisms of colistin-induced nephrotoxicity is oxidative stress, which can cause DNA damage, mitochondrial malfunction, and the formation of reactive oxygen species (ROS), increasing malondialdehyde (MDA), and attenuating glutathione (GSH) in renal tissue (6-8). Pathological investigation of the kidneys indicated considerable abnormalities in colistin-treated rats’ renal tissue, including tubular necrosis and interstitial inflammation. These findings imply that colistin-induced nephrotoxicity is associated with increased oxidative stress, which can result in impaired kidney function and pathological changes in renal tissue (9).

Recent studies have investigated the probable protecting properties of various natural products against colistin-induced nephrotoxicity including *Silybum marianum* (9), *Nigella sativa *(10), and alpha-lipoic acid (4). These findings suggest that natural products may have potential therapeutic applications in the management of colistin-induced nephrotoxicity.

*Crocus sativus*, commonly known as saffron, is a highly prized plant due to its culinary and medicinal uses. Saffron contains several bioactive compounds, including safranal, picrocrocin, and crocin which are responsible for its various pharmacological effects (11, 12). For centuries, saffron has been employed in traditional medicine to address a range of health issues such as asthma, allergies, and depression. Additionally, saffron is effective in the treatment of various diseases, such as Alzheimer’s disease, cardiovascular disease, and diabetes (13). Pharmacological studies have shown that saffron possesses anti-oxidant, anti-inflammatory (14, 15), anti-asthmatic (16, 17), antirheumatic (18), antidote (19), antidepressant (20), and neuroprotective (21, 22) properties. These findings suggest that saffron may have potential therapeutic applications in the management of various diseases.

Crocin, a major active constituent of saffron, has been shown to possess potent anti-oxidant properties (22, 23). Recent studies have investigated the potential ameliorative properties of crocin against various forms of nephrotoxicity, including drug-induced nephrotoxicity. For example, a study by Hosseinzadeh *et al.* investigated the protective effects of crocin against renal ischemia-reperfusion-induced oxidative damage in rats and found that crocin treatment could significantly reduce oxidative stress and improve kidney function (23). 

This study aimed to investigate the effectiveness of crocin in preventing colistin-induced nephropathy in a rat model, considering the properties of crocin and the mechanisms involved in the pathophysiology of the condition. The study is the first in the medical literature to assess the prophylactic effects of different doses of crocin (10, 20, and 40 mg/kg) in preventing nephropathy in the colistin-induced nephropathy model in rats, as determined through a literature search.

## Materials and Methods

***Chemicals***

The materials used were purchased from the following companies: Thiobarbituric acid (TBA), KCl, and phosphoric acid from Merck, Germany; 5, 50-dithiobis 2-nitrobenzoic acid (DTNB), tricarboxylic acid (TCA), and crocin from Sigma-Aldrich, the USA; Colistin from Exir Co, Iran.

***Animals***

The animals used were 36 healthy male Wistar rats weighing between 230 and 250 grams, housed in normal cages with a 12-hour light/dark cycle and kept at a temperature of 23±2 °C. Throughout the study period, the animals were provided with *ad libitum* access to food and water, except for the dehydration period. All animal experiments were carried out in accordance with Mashhad Pharmacy School Committee’s ethical guidelines (IR.MUMS.PHARMACY.REC.1400.042).

***Study protocol***

Six groups of male Wistar rats were employed (n=6):

1- Vehicle group (control): For ten days, rats in this group received normal saline (0.5 ml, IP). After the injection period, the animals in this group underwent a 24-hour dehydration period.

2- Crocin (40 mg/kg) group: This group was formed to study the effect of crocin alone on healthy animals. Crocin was administered intraperitoneally to rats at the highest dose every day for ten days. The animals of this group were dehydrated for 24 hr after the injection period ended.

3-Colistin (23 mg/kg, 7 days, IP) (dissolved in normal saline) group: This group was established to study colistin nephrotoxicity. Colistin was administered intraperitoneally to rats daily for seven days. The animals were dehydrated for 24 hr after the injection period. It is essential to note that pilot experiments (pathology, measuring renal MDA and GSH amounts, as well as serum urea and creatinine levels) were used to determine the appropriate dose between 10–30 mg/kg, but the duration and route of administration were chosen according to a previous study (24).

4- Crocin (10 mg/kg) (25)+ colistin (23 mg/kg) group. Crocin was dissolved in normal saline.

5- Crocin (20 mg/kg) (25)+ colistin (23 mg/kg) group.

6- Crocin (40 mg/kg) (25)+ colistin (23 mg/kg) group.

Groups 4–6 were formed to examine the protective impact of crocin on kidney damage triggered by colistin. For ten days, rats in these groups received an intraperitoneal injection of crocin 30 min before the injection of colistin. Furthermore, rats in these groups were given crocin for three days before the first injection of colistin. The animals in these groups were dehydrated for 24 hr after the injection period.

On the eleventh day of the trial, after the dehydration period was over, all rats were sacrificed and blood and right kidney samples were obtained. The samples were stored in liquid nitrogen first, then at -80 °C until further examination. In addition, for histological examinations, the left kidney was kept in 10% formaldehyde. There were six rats in each group.

***Measurement of kidney function***

Serum creatinine and BUN levels were measured by sending blood serum samples to the laboratory.

***The measurement of MDA level in kidney tissue***

MDA levels in the tissue rise as lipid peroxidation increases. In an acidic environment, MDA interacts with TBA to create a pink complex with maximal absorbance at 532 nm (26).

Initially, an average of 100 mg of tissue sample was separated, and a 10% homogenate was prepared using cold 1.15% KCL. The homogenate was then combined with 3 ml of 1% phosphoric acid and 1 ml of 0.6% TBA solution. The resulting mixture was then immersed in boiling water for 45 min. The cooled liquid was then vortexed for one minute with 4 ml of n-butanol to extract the colored complex. The organic phase was transferred to new tubes after centrifuging the mixture at 3000 g for 10 min, and the absorbance at 532 nm wavelength was measured for different samples. A standard curve for MDA concentrations ranging from 0 to 100 nmol/ml was drawn. Finally, the amount of MDA was expressed in nmol/g tissue (27).

***Measurement of GSH level in kidney tissue***

In an alkaline environment, free sulfhydryl groups react with DTNB reagent to form a colorful complex with maximum absorbance at 412 nm (28).

First, an average of 100 mg of each sample tissue was extracted, and a 10% homogenate was prepared using phosphate buffer at pH 7.4. After vortexing, the homogenized samples were mixed with 10% TCA in a 1:1 ratio and centrifuged at 3000 g for 10 min. The top phase was then separated and mixed with 2 ml of pH 8:8 phosphate buffer. The absorbance at 412 nm wavelength was measured for each sample after adding 0.5 ml of 0.04% DTNB reagent. To calculate the amount of GSH, a standard curve in the concentration range of 0–300 nmol/ml GSH was drawn, and its value was reported in terms of nmol/g of tissue (29).

***Investigation of pathological changes in kidney tissue***

The procedure involved the isolation of the left kidneys, which were then fixed in 10% neutral-buffered formalin. The kidney tissues were subsequently embedded in paraffin, dissected, and stained with hematoxylin and eosin. A pathologist examined the prepared histopathologic slides using a light microscope and assessed the differences among the groups (rated on a scale of 1 to 4, with 1 indicating normal and 4 indicating the most severe injury).

***Statistical analysis***

The statistical software program Prism 9 was used for the statistical calculations. The results are reported as Mean ± standard deviation (SD). One-way ANOVA test and post-test Tukey-Kramer were used for statistical comparison between different groups. *P*<0.05 was considered statistically significant. In addition, the non-parametric Kruskal-Wallis test was used to analyze pathological data, and the data were reported as median (interquartile range, IQR).

## Results

***Effect of crocin on blood BUN and creatinine levels in kidney damage caused by colistin***

**Figure 1:**
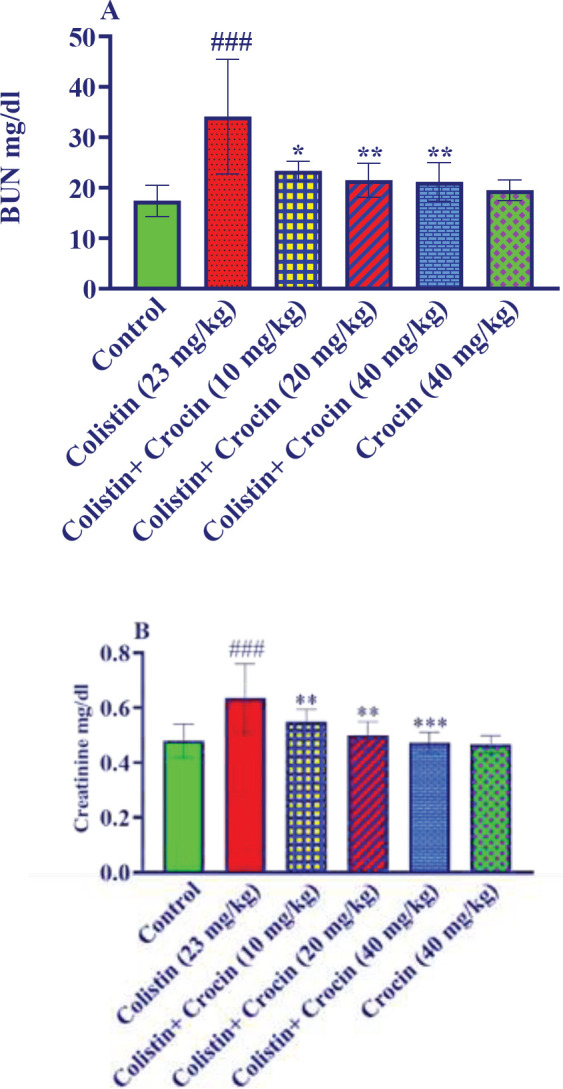
Effect of colistin and crocin on serum A: BUN and B: creatinine levels

Colistin administration (23 mg/kg) led to a significant increase in the amount of BUN in the serum of animals compared to the control group (*P*<0.001). Administration of crocin at doses of 10, 20, and 40 mg/kg along with colistin caused a significant reduction in BUN levels in comparison to the colistin group (*P*<0.05 for 10 mg/kg and *P*<0.01 for 20 and 40 mg/kg). However, administration of crocin at a dose of 40 mg/kg to healthy animals did not produce any significant changes compared to the control group (Figure 1A).

Compared to the control group, injection of colistin at a dose of 23 mg/kg resulted in a significant increase in kidney creatinine levels (*P*<0.001). However, co-administration of crocin at doses of 10, 20, and 40 mg/kg with colistin resulted in a significant reduction in creatinine levels compared to the colistin group (*P*<0.01 for 10 and 20 mg/kg, and *P*<0.001 for 40 mg/kg). Furthermore, administration of crocin at a dose of 40 mg/kg did not produce any significant changes in creatinine levels in healthy animals compared to the control group (as shown in Figure 1B).

***Effect of crocin on renal MDA and GSH levels in kidney damage caused by colistin***

**Figure 2:**
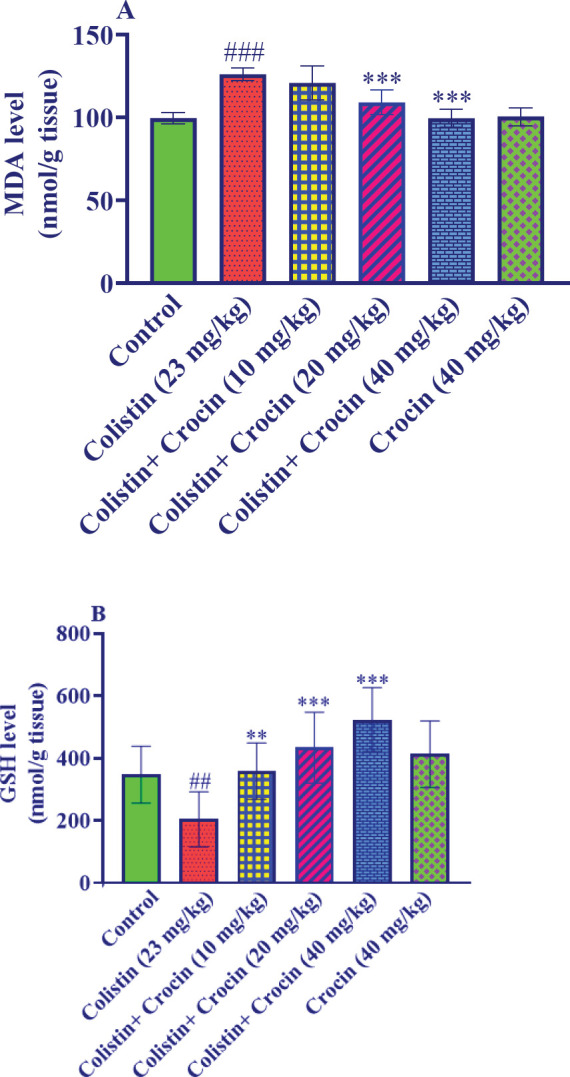
Effect of colistin and crocin on renal A: MDA and B: GSH levels

In this study, administering colistin at a dosage of 23 mg/kg resulted in a significant rise in kidney tissue MDA content when compared to the control group (*P*<0.001). When crocin was co-administered with colistin at a dose of 10 mg/kg, there was no significant impact on MDA levels in comparison to the colistin group. However, crocin at doses of 20 and 40 mg/kg led to a significant reduction in kidney tissue MDA content compared to the colistin group (*P*<0.001). Furthermore, there was no significant difference between the group that received crocin alone at a dose of 40 mg/kg and the control group (Figure 2A). 

In comparison to the control group, the injection of colistin at a dose of 23 mg/kg resulted in a significant reduction in kidney tissue GSH content (*P*<0.01). Crocin, at all three dosages (10, 20, and 40 mg/kg), however, induced a substantial increase in kidney tissue GSH content as compared to the colistin-only group (*P*<0.01 for 10 mg/kg and *P*<0.001 for 20 and 40 mg/kg). Figure 2B shows that there were no statistically significant differences between the group that received crocin at a dosage of 40 mg/kg alone and the control group.

***Effect of crocin on renal histopathological alterations caused by colistin***

**Figure 3:**
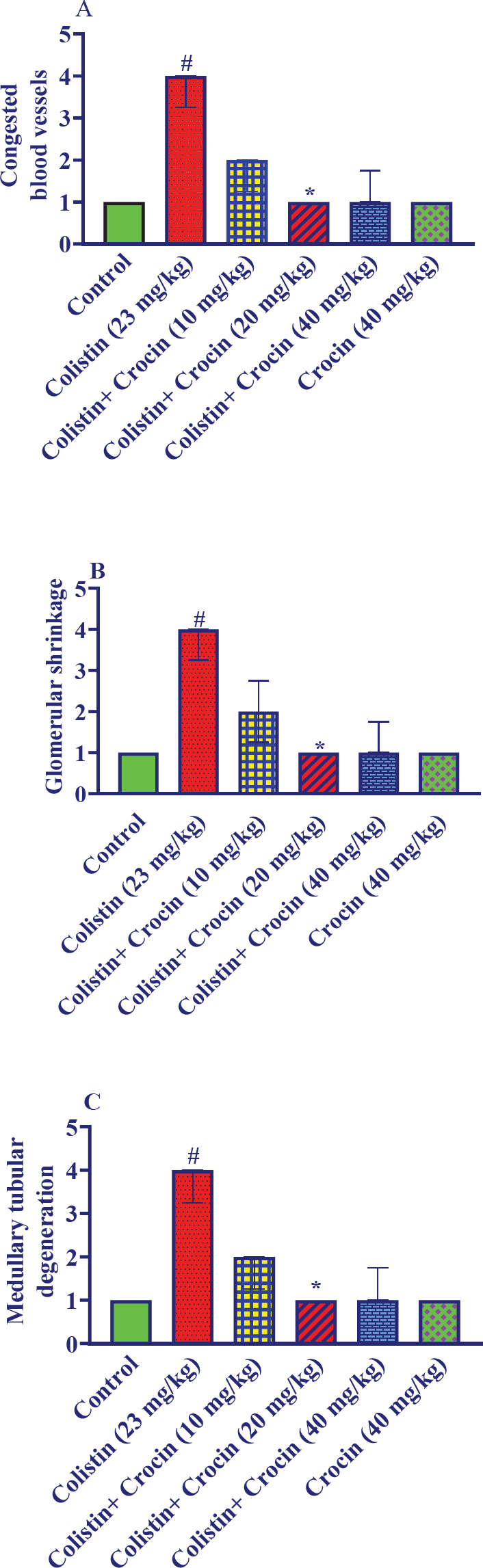
Effect of colistin and crocin on renal histology. A: Congested blood vessels, B: Glomerular shrinkage, C: Medullary tubular degeneration

**Figure 4:**
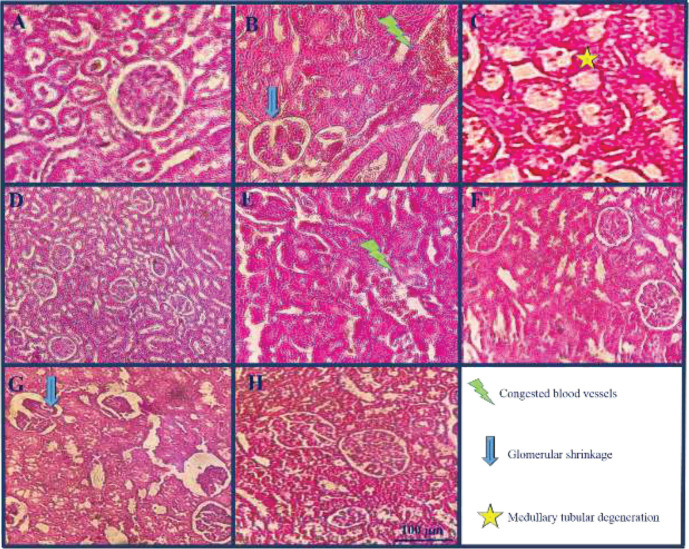
Photomicrograph of the rat kidney sections staining with hematoxylin and eosin. A: Normal glomeruli, B: Congested blood vessel and glomerular shrinkage, C: Medullary tubular degeneration, D: Control group, E: Colistin plus crocin (10 mg/kg) group, F: Colistin plus crocin (20 mg/kg) group, G: Colistin plus crocin (40 mg/kg) group, and H: Crocin (40 mg/kg) group (40X)

Receiving colistin resulted in congested blood vessels, glomerular shrinkage, and medullary tubular degeneration (*P*<0.05) (Figures 3 and 4). Co-administration of crocin 20 mg/kg along with colistin significantly ameliorated these alterations (*P*<0.05). The concurrent administration of crocin 10 and 40 mg/kg besides colistin reduced congested blood vessels, glomerular shrinkage, and medullary tubular degeneration, although it was not significant. Additionally, there were no significant differences observed between the control group and the group that received a dosage of 40 mg/kg of crocin alone.

Colistin (23 mg/kg) and crocin (10, 20, and 40 mg/kg) were administered intraperitoneally to the animals. The data are presented as Mean±SD (n=6). ANOVA and Tukey-Kramer post-test were used to assess statistical differences. ###*P*<0.001 compared to the control, ***P<0.001, **P<0.01, and *P<0.05 compared to colistin

BUN: blood urea nitrogen

Colistin (23 mg/kg) and crocin (10, 20, and 40 mg/kg) were administered intraperitoneally to the animals. The data are presented as Mean±SD (n=6). ANOVA and Tukey-Kramer post-test were used to assess statistical differences. ###*P*<0.001 and ##*P*<0.01compared to the control, ***P<0.001 and **P<0.01 compared to colistin

MDA: malondialdehyde; GSH: glutathione

Colistin (23 mg/kg) and crocin (10, 20, and 40 mg/kg) were administered intraperitoneally to the animals

ANOVA test and non-parametric Kruskal-Wallis test were used to check the statistical difference. #*P*<0.05 compared to the control and *P<0.05 compared to colistin

## Discussion

Colistin, a medication that had previously lost favor due to reports of nephrotoxicity and neurotoxicity, has recently attracted attention due to the rise of multidrug-resistant gram-negative bacteria (30). The reduction of these side effects would increase the therapeutic value of colistin and make it possible to administer higher doses with greater effectiveness (31). Therefore, developing preventative strategies to lessen colistin-induced nephrotoxicity is crucial. It has been discovered that the pathogenesis of colistin-induced nephrotoxicity is complicated, involves oxidative stress, interferes with renal function, and begins after colistin accumulates in the kidneys (32). Crocin, an active component of *Crocus sativus*, has been demonstrated to prevent and ameliorate nephrotoxicity through different mechanisms including the lessening of oxidative stress, and improving renal function (33, 34). Therefore, the present study aimed to investigate the potential protective effects of crocin (10, 20, and 40 mg/kg, IP) against colistin-induced nephrotoxicity in rats. 

An earlier study disclosed that colistin (1000,000 IU/kg/day, 10 days, p.o.) induced nephrotoxicity can manifest as an increase in serum creatinine and BUN in rodents (32). Another study found that receiving colistin (10 mg/kg, twice a day, 7 days, IP) resulted in augmented levels of serum creatinine and BUN in rats (35). In line with previous studies, the intraperitoneal injection of colistin (23 mg/kg, 7 days, IP) elevated BUN and creatinine levels in rats’ serum samples. On the other hand, concurrent administration of crocin (10, 20, and 40 mg/kg, 7 days, IP) reversed the alterations induced by colistin in creatinine and BUN levels. Furthermore, crocin (20 mg/kg, 21 days, p.o.) could decrease BUN and creatinine levels in rats with streptozotocin-induced nephropathy (36). In another similar study, crocin (20 mg/kg, 8 weeks, p.o.) reduced serum creatinine levels, BUN, and proteinuria with associated growth in urinary creatinine clearance in rats with diabetic nephropathy (33).

There is likely to be a positive correlation between serum creatinine/BUN and MDA or a negative correlation with GSH. As renal function deteriorates and damage accumulates, oxidative stress also aggravates the kidneys. Besides, higher oxidative stress further worsens renal impairment (37). Moreover, it has been revealed that oxidative stress contributes to the development of colistin-induced nephropathy (38). In rats, intraperitoneal injection of colistin (300,000 IU/kg, 6 days) has been demonstrated to induce oxidative stress in kidney tissue, as indicated by an upsurge in renal MDA levels and a reduction in GSH levels (31). Likewise, the results of an *in vivo* study indicated that the administration of colistin increased renal oxidative stress by enhancing MDA amounts and attenuating GSH, superoxide dismutases, catalase, and glutathione peroxidase levels (38). Our findings also revealed that colistin triggered oxidative stress in kidney tissue which was disclosed by an elevation in MDA amounts and a reduction in GSH levels. However, co-administration of crocin with colistin reduced oxidative stress in kidney tissues. Our results reinforced previous investigations that reported crocin ameliorated nephropathy by its anti-oxidant effect in several experimental nephrotoxicity models, including tartrazine-induced nephrotoxicity (39), carbon tetrachloride-induced renal toxicity (40), and cisplatin-induced renal oxidative stress (34). In summary, serum creatinine, BUN, MDA, and GSH levels can serve as mutual indicators of each other in colistin-caused nephropathy. Controlling any one of these factors can help modulate the others for better renal health.

The pathogenesis of kidney disorders involves the contribution of oxidative stress (41). For instance, oxidative stress stimulates the production of collagen and fibronectin, leading to glomerulosclerosis and tubulointerstitial fibrosis that impairs glomerular filtration. Oxidative stress may trigger calcium phosphate deposition in the kidneys by enhancing calcium reabsorption in damaged tubules. These mineral deposits can obstruct tubules and accelerate damage (42, 43). The pathological alterations in kidney tissues were confirmed by previous studies; for example, a study reported that colistin (750.000 IU/kg/day, 7 days, IP) for seven days resulted in acute tubular necrosis, tubular injury, interstitial inflammation, and medullar congestion in rats (9). Another study stated that colistin accumulative dose caused severe intratubular hemorrhage, protein cast, degenerated tubular epithelium with vacuolated cytoplasm, and pyknotic nuclei in rats (44). Our results also showed that colistin intraperitoneal injection led to congested blood vessels, glomerular shrinkage, and medullary tubular degeneration that was amended by crocin concurrent administration. Furthermore, Rezaee-Khorasany *et al.* reported that crocin (20 mg/kg) could ameliorate the histopathological alterations induced by ethanol in renal tissues in rats (25). Likewise, crocin could amend pathological alterations in the doxorubicin-induced nephrotoxicity model in rats (45).

## Conclusion

According to the findings of the study, colistin-induced renal damage is associated with oxidative stress, as demonstrated by higher renal MDA and a decrease in GSH levels. It also raises serum BUN and creatinine levels. Crocin, on the other hand, enhances its renoprotective properties against colistin-induced nephrotoxicity by reducing oxidative stress, metabolic changes, and histological damage in rat kidneys. Therefore, crocin may be a viable medication for preventing the negative side effects of colistin therapy.

## Authors’ Contributions

H H and BM R were supervisors, designed the work, revised it critically for important intellectual content, and approved the version to be published. R R performed the experiment, and M GR helped in performing the experiments and wrote the proposal and manuscript.

## Data Availability Statements

The data that support the findings of this study are available from the corresponding author upon reasonable request.

## Funding

This research was supported by the Vice-Chancellor of Research, Mashhad University of Medical Sciences (No:4000249).

## Conflicts of Interest

The authors declare that they have no conflicts of interest.
